# Supplementary material for: Benchmarking of quantum protocols
Source: Sci Rep. 2022 Mar 28;12:5298. doi: 10.1038/s41598-022-08901-x (PMC8964774; doi:10.1038/s41598-022-08901-x)
Supplement: Supplementary file 1 — Supplementary Information. [file 41598_2022_8901_MOESM1_ESM.pdf]

Benchmarking of Quantum Protocols

Appendix A
Derivation of $Pr(A|B)$ and $Pr(A|C)$

In this appendix, we calculate the two probabilities $Pr(A|B)$ and $Pr(A|C)$. First of all, we note that $Pr(A|B) = 1 - Pr(A'|B)$ and $Pr(A|C) = 1 - Pr(A'|C)$, where A' is the event that all measurement outcomes are zero. First we consider the ideal case where there is no noise. It has been shown in [1] that in this case $Pr(A'|B) = 2/N$. To obtain $Pr(A'|C)$, note that the quantum state after loss of one particle is

$$\rho_{\text{loss}} = \frac{N-1}{N}|W\rangle\langle W|_{N-1} + \frac{1}{N}|\vec{0}\rangle\langle\vec{0}|_{N-1} \tag{1}$$

Then, $Pr(A'|C)$ is given by

$$\begin{aligned} Pr(A'|C) = \text{Tr}[\rho_{\text{loss}}(\mathbf{1}_{SR} \otimes |\vec{0}\rangle\langle\vec{0}|_{N-3})] = \\ (\frac{N-1}{N})(\frac{2}{N-1}) + \frac{1}{N} = \frac{3}{N} \end{aligned} \tag{2}$$

In the above equation, $\mathbf{1}$ denotes identity matrix.

Next, we consider the case where generation and distribution of W state is not ideal. We assume dephasing noise model with the noise parameter v as follows:

$$\Lambda(\rho) = v\rho + (1-v)\sigma_z\rho\sigma_z, \tag{3}$$

where σ_z is the Pauli Z matrix. The probability $Pr(A'|B)$ can be expressed as

$$Pr(A'|B) = \text{Tr}[(\Lambda^{\otimes N}|W\rangle\langle W|_N)(\mathbf{1}_{SR} \otimes |\vec{0}\rangle\langle\vec{0}|_{N-2})], \tag{4}$$

We note that the term $\mathbf{1}_{SR} \otimes |\vec{0}\rangle\langle\vec{0}|_{N-2}$ is a diagonal matrix. Since dephasing noise only affects the non-diagonal elements of the state $|W\rangle\langle W|_N$, we can simply conclude that

$$\begin{aligned} \text{Tr}[(\Lambda^{\otimes N}|W\rangle\langle W|_N)(\mathbf{1}_{SR} \otimes |\vec{0}\rangle\langle\vec{0}|_{N-2})] = \\ \text{Tr}[|W\rangle\langle W|_N(\mathbf{1}_{SR} \otimes |\vec{0}\rangle\langle\vec{0}|_{N-2})] = \frac{2}{N}. \end{aligned} \tag{5}$$

From the above equation, we can simply conclude that $Pr(A'|C)$ also does not change compared to the noiseless case.

Appendix B
VBQC protocol

In this appendix, the steps of the VBQC protocol proposed in [2] are outlined. We assume that the server has three qubits. This protocol consists of N runs, where d runs are computation runs and $t = N-d$ runs are test runs. The client chooses uniformly at random the test runs. In what follows, $\mathcal{M}^{\alpha}$ denotes a measurement in basis $|\pm_\alpha\rangle = (|0\rangle \pm e^{i\alpha}|1\rangle)/\sqrt{2}$. The outcome will be zero for the projector $|+_\alpha\rangle\langle +_\alpha|$ and 1 for $|-_\alpha\rangle\langle -_\alpha|$. The inputs of the protocol are as follows:

a) $x \in \{0, 1\}$

b) ϕ_1, ϕ_2, ϕ_3 from the set $C = \{k\pi/8\}, k \in [0, 7]$

In each test run, the following steps are performed:

1) Client and server establish three entangled links between them. We denote server's qubits by q_1, q_3 and q_5. Client's qubits entangled with q_1, q_3 and q_5 are denoted by q_2, q_4 and q_6, respectively.

2) Server applies CZ on q_1 and q_3. Then, server applies CZ on q_3 and q_5.

3) Client randomly chooses $u \in \{1,2\}$.

4) Client randomly chooses θ_1, θ_2, and θ_3 from the set C.

5) if $u=1$, client applies $\mathcal{M}^{-\theta_1}$ on q_2, with the result denoted by g_1. The client applies $\mathcal{M}^{-\theta_3}$ on q_6, with the result denoted by g_3. The client measures q_4 in the standard basis, with the result denoted by d_2.

6) If $u=2$, client applies $\mathcal{M}^{-\theta_2}$ on q_4, with the result denoted by g_2. Client measures q_2 and q_6 in the standard basis, with the results denoted by d_1 and d_3, respectively.

7) Client randomly chooses r_1, r_2 and r_3 from the set $\{0,1\}$.

8) If $u=1$, client assigns $\delta_1 = \theta_1 + (r_1 + d_2 + g_1)\pi$. Otherwise, client randomly chooses δ_1 from the set C. Client sends δ_1 to server. Server applies $\mathcal{M}^{\delta_1}$ on q_1 and sends the result, denoted by b_1, to client.

9) If $u=1$, client randomly chooses δ_2 from the set C. Otherwise, client assigns $\delta_2 = \theta_2 + (r_2 + d_1 + d_3 + g_2)\pi$. Client sends δ_2 to the server. The server applies $\mathcal{M}^{\delta_2}$ on q_3 and send the result, denoted by b_2, to client.

10) If $u=1$, client assigns $\delta_3 = \theta_3 + (r_3 + d_2 + g_3)\pi$. Otherwise, client randomly chooses δ_1 from the set C. Client sends δ_1 to server. Server applies $\mathcal{M}^{\delta_3}$ on q_5 and sends the result, denoted by b_3, to client.

11) If $u=1$, client verifies the test round if $r_1 = b_1$ and $r_3 = b_3$. Otherwise, client verifies the test round if $r_2 = b_2$.

In each computation run the following steps are performed:

1) Client and server establish three entangled links between them. We denote server's qubits by q_1, q_3 and q_5. Client's qubits entangled with q_1, q_3 and q_5 are denoted by q_2, q_4 and q_6, respectively.

2) Server applies CZ on q_1 and q_3. Then, Client applies CZ on q_3 and q_5.

3) Client randomly chooses θ_1, θ_2, and θ_3 from the set C.

4) Client applies $\mathcal{M}^{-\theta_1}$, $\mathcal{M}^{-\theta_2}$, and $\mathcal{M}^{-\theta_3}$ to q_2, q_4 and q_6, respectively, with the results assigned to g_1, g_2 and g_3, respectively.

5) Client randomly chooses r_1, r_2 and r_3.

6) Client sends $\delta_1 = \phi_1 + \theta_1 + (x + r_1 + g_1)\pi$ to server. Server applies $\mathcal{M}^{\delta_1}$ to q_1 and sends the result, b_1 to client.

7) Client sends $\delta_2 = (-1)^{b_1+r_1}\phi_2 + \theta_2 + (r_2 + g_2)\pi$ to server. Server applies $\mathcal{M}^{\delta_2}$ to q_3 and sends the result, b_2 to client.

8) Client sends $\delta_3 = (-1)^{b_2+r_2}\phi_3 + \theta_3 + (b_1 + r_1 + r_3 + g_3)\pi$ to server. Server applies $\mathcal{M}^{\delta_3}$ to q_5 and sends the result, b_3 to client.

9) Client considers $b_3 \oplus r_3$ as the output.

If the number of failed test runs is larger than a threshold the protocol aborts.

Appendix C
QDS protocol

In this appendix, the QDS protocol proposed in [3] is explained in details. This protocol has three parties, namely, Alice, Bob, and Charlie. Alice sends the signed message to Bob. Bob authenticates the message and forwards it to charlie. The protocol is consisted of two parts: distribution stage, and messaging stage.

In the distribution stage, for each possible message $m=0$ or $m=1$, key generation protocol (KGP) is performed by Alice-Bob and Alice-Charlie separately. The number of transmitted qubits in KGP is denoted by N. In [3], weak coherent states with decoy states technique are used in the KGP. Here, we assume KGP is performed using single photon states. To perform KGP between Alice and Bob, Bob randomly chooses from the four states $|0_Z\rangle$,$|1_Z\rangle$ (Z basis), and $|0_X\rangle = 1/\sqrt(2)(|0_Z\rangle + |1_Z\rangle)$, $|1_X\rangle = 1/\sqrt(2)(|0_Z\rangle - |1_Z\rangle)$ (X basis) and sends the quantum states to Alice. The X and Z bases are selected with probabilities $P_X \geq 0.5$ and $P_Z = 1 - P_X$, respectively. Alice randomly chooses her measurement basis X or Z with probabilities P_X and P_Z, respectively. Then, Alice and Bob perform key sifting to obtain two strings with matched bases. Alice and Bob's key strings are denoted by A_m^B and $K_m^B = \{X_m^B, Z_m^B\}$, respectively. Here, X_m^B and Z_m^B denote Bob's key in X and Z bases respectively. Similarly, Alice and Charlie perform KGP and generate bit strings A_m^C and $K_m^C = \{X_m^C, Z_m^C\}$.

Next, Bob randomly selects a fraction r of X_m^B, denoted by V_m^B to estimate the error with Alice. The estimated error rate is denoted by e_X^{BA}. Then, he randomly chooses half of the remaining bits and sends them to Charlie. The forwarded and left strings are denoted by $X_m^{B,\text{forward}}$ and $X_m^{B,\text{keep}}$, respectively. Similarly, Charlie does the same procedure, and obtains e_X^{CA}, $X_m^{C,\text{forward}}$ and $X_m^{C,\text{keep}}$. At the end of the distribution stage, Bob and Charlie have the strings $R_m^B = \{X_m^{B,\text{keep}}, X_m^{C,\text{forward}}\}$ and $R_m^C = \{X_m^{C,\text{keep}}, X_m^{B,\text{forward}}\}$, respectively. Moreover, Alice has two strings $\acute{A}_m^B$ and $\acute{A}_m^C$. The length of these four strings is represented by l.

In the messaging stage, Alice sends (m, S_m) to Bob, where $S_m = \{\acute{A}_m^B, \acute{A}_m^C\}$. Bob checks the mismatches between R_m^B and S_m. He will accept the message and forward it to Charlie if there are fewer than $s_a l$ mismatches in both halves of his string, where $0 < s_a < 0.5$ is a threshold. Similarly, Charlie will accept the message if there are fewer than $s_v l$ mismatches in both halves of his key, where $0 < s_v < s_a < 0.5$.

In [3], the thresholds s_a and s_a are chosen to be

$$s_a = \frac{P_E + 2e_X^U}{3} \qquad (6)$$
$$s_v = \frac{2P_E + e_X^U}{3}.$$

In the above equations, $e_X^U = \max\{e_{X,B}^U, e_{X,C}^U\}$, where $e_{X,B}^U$ and $e_{X,C}^U$ are upper bounds on error rate in X basis. The parameter $e_{X,B}^U$ and $e_{X,C}^U$ can be obtained using e_X^{BA} and e_X^{CA}, respectively. To this aim, Serfling inequality [4] is applied to bound the actual error rate. The parameter P_E is given by

$$h(P_E) = 1 - h(\phi_X^U), \qquad (7)$$

where $h(.)$ is binary entropy function, and ϕ_X^U is the upper bound on the phase error rate in the X basis. This parameter is obtained using the estimated bit error rate in the Z basis, as in [5].

The security of the protocol is characterized by P_{abort}, P_{for}, P_{rep}. The parameter P_{abort} is given by

$$P_{\text{abort}} = 2\varepsilon_{PE}, \qquad (8)$$

where ε_{PE} is the failure probability in calculating the upper bounds for error rates. P_{rep} can be expressed as

$$P_{\text{rep}} = 2e^{-0.5l(s_a - s_v)^2}. \qquad (9)$$

Finally, the parameter P_{for} is given by

$$P_{\text{for}} = a + \epsilon_F + 8\varepsilon_{PE}, \qquad (10)$$

where

$$\epsilon_F = \frac{1}{a}(\varepsilon + 2^{(-l(1-h(\phi_X^U)-h(s_v))}). \qquad (11)$$

In the above equation, a and ε are small constants.

Appendix D
NetSquid implementation

In this appendix, we provide some details on NetSuid simulations. The code repository for all NetSquid simulations performed in this work can be found in [6]. A README file is included for each protocol, which provides a general description of the protocol, protocol steps, protocol parameters, and other necessary details.

References

[1] V. Lipinska, G. Murta, and S. Wehner, "Anonymous transmission in a noisy quantum network using the w state," *Physical Review A*, vol. 98, no. 5, p. 052320, 2018.

[2] E. Kashefi, D. Leichtle, L. Music, and H. Ollivier, "Securing quantum computations in the NISQ era," *arXiv preprint arXiv:2011.10005*, 2020.

[3] R. Amiri, P. Wallden, A. Kent, and E. Andersson, "Secure quantum signatures using insecure quantum channels," *Physical Review A*, vol. 93, no. 3, p. 032325, 2016.

[4] R. J. Serfling, "Probability inequalities for the sum in sampling without replacement," *The Annals of Statistics*, pp. 39–48, 1974.

[5] Z. Zhang, Q. Zhao, M. Razavi, and X. Ma, "Improved key-rate bounds for practical decoy-state quantum-key-distribution systems," *Physical Review A*, vol. 95, no. 1, p. 012333, 2017.

[6] C.-T. Liao, https://github.com/LiaoChinTe/netsquid-simulation, 2021.
